# The Effect of Guided Imagery on Stress and Fatigue in Patients with Thyroid Cancer Undergoing Radioactive Iodine Therapy

**DOI:** 10.1155/2013/130324

**Published:** 2013-11-24

**Authors:** Mi Hye Lee, Dong-Hee Kim, Hak Sun Yu

**Affiliations:** ^1^Department of Nursing, College of Nursing, Pusan National University, Yangsan-si, Gyeongsangnam-do 626-870, Republic of Korea; ^2^Department of Parasitology, School of Medicine, Pusan National University, Yangsan-si, Gyeongsangnam-do 626-870, Republic of Korea; ^3^Immunoregulatory Therapeutics Group in Brain Busan 21 Project, Busan Metropolitan City, Yeonje-gu 611-735, Republic of Korea

## Abstract

This study was conducted to evaluate the effects of guided imagery on stress and fatigue in patients undergoing radioactive iodine therapy after thyroidectomy in Korea. Participants were 84 individuals (44 for experimental group and 40 for control group) with thyroid cancer. The experimental group listened to a guided imagery CD once a day for 4 weeks. Global Assessment of Recent Stress and Revised Piper Fatigue Scale were self-administered, and heart rate variability was measured at three time points; prior to intervention (T1), just before intervention (T2) and 1 week later after intervention (T3). Heart rate variability was consisted of Standard Deviation of all NN interval (SDNN), Total Power (TP), Low Frequency (LF), and High Frequency (HF). There were significant decreases in stress (*F* = 28.45, *P* < 0.001) and fatigue (*F* = 26.17, *P* < 0.001) over time in the experimental group compared to the control group. Heart rate variability changed over time in the experimental group relative to the control group; SDNN (*F* = 6.68, *P* = 0.002), TP (*F* = 5.29, *P* = 0.006), LF (*F* = 4.58, *P* = 0.012), and HF (*F* = 3.71, *P* = 0.026). From the results of this study guided imagery can be recommended as an effective intervention to thyroid cancer patients with stress and fatigue.

## 1. Introduction

Thyroid cancer, which is the most common endocrine cancer, has increased in incidence worldwide over the last few decades. The average increase of thyroid cancer rates during the 30-year period 1973–2002 worldwide was 58.1%, with 48.0% among males and 66.7% among females [1]. Thyroid cancer caused from 24,000 in 1990 to 36,000 deaths for 20 age groups in 2010 globally [2]. According to the Korean National Cancer Information Center, thyroid cancer accounts for 17.8% of all cancers, ranking it first among all cancers based on incidence rates [3]. Additionally, the incidence of thyroid cancer has increased from 3.6 per 100,000 in 1973 to 8.7 per 100,000 in 2002 in the United States [4]. Surprisingly, thyroid cancer mortality has not decreased but is increasing, despite earlier diagnosis and better treatment [5]. 

The most effective management of thyroid cancer is thyroidectomy followed by radioactive iodine treatment and TSH-suppression therapy. Radioactive iodine treatment usually consists of I-131 administered orally in tablet form, which destroys any cancer cells left behind after thyroidectomy. For successful radioactive iodine treatment, patients have to stop taking thyroid hormone replacement medication for 2–4 weeks so that they have a high level of thyroid stimulating hormone. Patients also need to have a low or no iodine diet for two weeks before treatment. Because radioactive iodine treatment will make the patients slightly radioactive for about a week, the patients must also maintain a safe distance from other people, including family members, friends, and medical staffs, necessitating that they remain an isolated place [1].

Individuals newly diagnosed with cancer often undergo emotional stress, which increases demands on cognitive resources and leads to fatigue. Additionally, the overall levels of stress increase as fatigue causes reduced effectiveness of daily function [6]. Moreover, patients waiting for surgery or medical treatment are burdened with a significant amount of stress and anxiety [7, 8]. Patients with thyroid cancer show significantly increased perceived stress scores when compared to the normal population and similar or higher scores than other oncologic patients [9]. Therefore, health care providers should help the population with thyroid cancer alleviate stress and fatigue. 

Pharmacological and nonpharmacological strategies can be recommended to treat fatigue and stress [10]. Since the causes or consequences of these symptoms are related to psychophysiological and psychobehavioral nature, a mind-body intervention such as guided imagery can be considered for management of these symptoms [11, 12]. Richardson et al. reported that patients diagnosed with breast cancer using guided imagery to cope with radiation therapy reported lower stress levels and more energy [13]. Moreover, patients using guided imagery intervention reported better self-care [14] and enhanced comfort levels during treatment [15]. Recently, Serra et al. evaluated the impact of guided imagery on patients receiving radiation therapy for breast cancer. Overall, 86% patients in their study described the guided imagery intervention as helpful and stated that they would recommend the intervention to others. Moreover, decreased respiration rate, pulse rate, systolic blood pressure, and diastolic blood pressure were observed, while skin temperature increased, indicating a decrease in the sympathetic response [16].

Nevertheless, relatively few studies of the effectiveness of the guided imagery intervention have been conducted among the cancer population in spite of the effects of the guided imagery intervention on cancer-related symptoms in previous studies. Moreover, to the best of our knowledge, no studies have examined the impact of guided therapy on fatigue and stress among the thyroid cancer. Therefore, this study was conducted to assess the effects of guided imagery intervention on distress and fatigue among the population with thyroid cancer undergoing radioactive iodine therapy after thyroidectomy in Korea. 

## 2. Methods

### 2.1. Design

This study consisted of a pre- and posttest consecutive experimental design to investigate the effects of guided imagery on distress and fatigue in patients being treated for thyroid cancer relative to a control group. 

### 2.2. Participants

**Figure 1:**
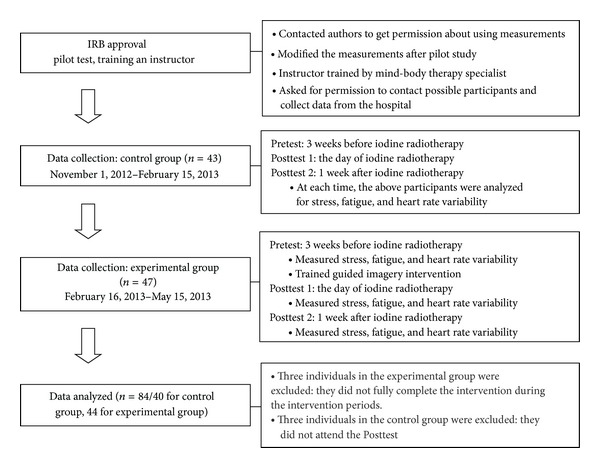
Flow chart of data collection.

The study sample consisted of patients scheduled for iodine radiotherapy after thyroidectomy at an institute of radiological and medical science specializing in cancer treatment in Korea. Inclusion criteria were 18 years and older, scheduled for the first iodine radiotherapy, normal EKG, and blood pressure. Exclusion criteria were having metastasis to other organs and any radiotherapy, chemotherapy history, diagnosed cardiovascular diseases, endocrine diseases, and sleep disorders. Previous studies have reported effect sizes between 0.29 and 6.64 [17]. Assuming an effect size *f* = 0.4 at the 5% level of significance with 80% power using repeated measures analysis of variance, a minimum of 62 subjects were required [18]. Based on an estimated follow-up loss, we initially targeted 92 patients. Among these patients, 90 signed the informed consent to be included in this study. These patients were then assigned to either an experimental group (*n* = 47) that received guided imagery therapy or a control group (*n* = 43) that received no guided imagery therapy. Three individuals in the experimental group were excluded because they did not fully complete the intervention during the intervention periods, while three individuals in the control group were excluded since they did not want to participate continuously in this study after baseline measure. As a result, data from a total of 84 participants were used for analysis.  Figure 1 shows the flow chart of participants of this study.

### 2.3. Intervention

The intervention for the study was guided imagery that was used by participants in the experimental group during a 4-week treatment period. Intervention was considered to have started when the participants visited a department of nuclear medicine to attend radioactive iodine therapy preparation education, which was usually 3 weeks before the radioactive iodine therapy began. The length of time for the guided imagery CD and the period for applying the intervention were based on previous studies [19, 20]. Specifically, treatment consisted of viewing a 13-minute imagery CD once a day before sleep for 4 weeks. The intervention was applied mostly at home and during 2 days at the hospital while patients received radioactive iodine therapy.

Participants in experimental groups were provided with a guided imagery CD and CD player. Participants in the experimental group received intervention instructions and were asked to practice once a day for 4 weeks. Participants in the experimental group were taught to imagine a peaceful scene when they visited a department of nuclear medicine. In addition, they were exposed to a variety of senses when listening to the contents of the CD (e.g., the participant listened to the sound of waterfall and smelled a rose) during face-to-face sessions in which intervention instruction was provided by the instructor. The participants were subsequently asked to rate how much they were able to image using a 7 point likert scale of 1 (very well) to 7 (not at all). If the participant rated more than 2 or wanted to experience the sense repeatedly, the instructor allowed it.

Participants in the control group were provided general information about radioactive iodine therapy.

### 2.4. Measurement

The Global Assessment of Recent Stress Scale (GARS scale) developed by Linn was used to measure stress [21]. The original English version of the instrument used in this study was translated to Korean and tested among cancer patients by Koh [22]. The Korean version of the GARS scale has been widely applied in Korea. This instrument consists of 8 typical Likert items ranging from 0 (not at all true of me) to 9 (extremely true of me). The Cronbach's alpha of the instrument reported by Koh was 0.81 [22]. In this study, the Cronbach's alpha was 0.94.

 Fatigue was assessed using the Revised Piper Fatigue Scale developed and revised by Piper et al. [23]. The original English version of the instrument used in this study was translated to Korean by Lee, who also reported that the Korean version of the instrument was valid and reliable in Korean women with cancer [24]. The Korean version of the revised PFS consists of a total of 19 items measuring four dimensions of subjective fatigue: behavioral/severity, affective meaning, sensory, and cognitive. Each item was measured on a 0 to 10 numeric rating scale, with opposing word anchors such as “not at all” to “a great deal.” The Cronbach's alpha of the instrument reported by Lee was 0.93 [24], and that for the present study was 0.98.

Since there was no possibility of directly measuring participant stress and fatigue, HRV was applied. Heart rate variability is a measure of continuous interplay in the autonomic nervous system including the sympathetic nervous system (SNS) and parasympathetic nervous system (PSNS) [25]. Thayer et al. conducted meta-analysis of HRV as a marker of stress and highlighted the importance of HRV as a potential marker of stress [26]. Additionally, Segerstrom and Nes recommended HRV as a possible index measuring fatigue [27]. To determine HRV, we used an SA-3000P cardio wave analyzer (Medicore Co., Ltd., Korea). The standard deviation of all NN interval (SDNN), total power (TP; ≤0.40 Hz), low-frequency (LF; 0.04–0.15 Hz), and high-frequency (HF; 0.15–0.40 Hz) were determined for evaluation in this study. In general, SDDN is represented by changes in heart rate over 5 min. SDDN considers total variability since it reflects all cycle components responsible for variability. HF represents the activity of the parasympathetic nerve system, while LF reflects a mixture of both the sympathetic and parasympathetic nerve systems [28]. We measured HRV parameters for 5 minutes in a quiet, private room with a temperature of 22–25°C after 10 minutes of rest. 

The instruments were checked for content validity by one registered nurse, one professor from the nursing school and one guided imagery therapist with a Ph.D. Two pilot studies were conducted prior to this study to enhance the comprehension of the questionnaire items, assess the validity and reliability of all measures, and determine the time required to administer the questionnaire and apply HRV among the thyroid cancer population.

### 2.5. Procedures

All patients scheduled to attend radioactive iodine therapy preparation education between 15 November, 2012 and 5 April, 2013 were potential participants. A research assistant contacted all patients at the department of nuclear medicine, explained the purpose of this study, and asked them to voluntarily sign a form once they indicated their willingness to participate. Global assessment of recent stress and revised Piper fatigue scale were self-administered, and heart rate variability was measured at three time points: prior to intervention (T1), immediately before intervention (T2), and 1 week after intervention (T3), by the research assistant. Heart rate variability consisted of the Standard Deviation of all NN interval (SDNN), Total Power (TP), Low Frequency (LF), and High Frequency (HF) data. T2 and T3 were set based on patients undergoing radioactive iodine therapy experiencing high levels of stress and fatigue during these periods [29–31].

The instructor sent text messages at the same time every day to remind participants to undergo the imagery and called the patients on a weekly basis to check their progress. Data were collected from November 15, 2012 to May 03, 2013. 

### 2.6. Data Analysis

Collected data were subjected to frequency, percentage, mean, and standard deviation analysis, an *χ* ^2^ test as well as repeated measures ANOVA using SPSS version 18.0 for Windows (SPSS, Inc, IBM Company, Chicago, Il, USA). Greenhouse-Geisser correlation epsilon was used in cases where the Mauchly's sphericity test for normality was violated to correct the degrees of freedom. Two-tailed *P* values < 0.5 were considered significant.

### 2.7. Ethical Consideration

Written permission for the study was received from the Dongnam Institute of Radiological and Medical Science. Participants agreed to the purpose of this study, voluntarily signed a form, and participated in the study. Participation was entirely voluntary. Individual informed consent was obtained from each participant to ensure anonymity. A written summary of the study was given to those willing to participate in this study. Participants were free to refuse to participate or withdraw from the study at any time and were informed that only the aggregate data would be reported. 

## 3. Results

### 3.1. Homogeneity between the Experimental and Control Groups

Homogeneity testing revealed that all general characteristics and outcome variables including stress, fatigue, and heart rate variability consisting of SDNN, TP, LF, and HF were equally distributed between the experimental and control groups (Tables 1 and 2).

### 3.2. Stress

There were significant differences over time in the stress of the experimental group compared to the control group (*F* = 28.45, *P* < 0.001). Specifically, the experimental group showed decreased stress scores from the baseline to 3 weeks, and this decline continued from 3 to 4 weeks. Conversely, there was an increase in the stress score of the control group from the baseline to 3 weeks, which was maintained from 3 to 4 weeks (Table 3).

### 3.3. Fatigue

There were significant differences over time in the fatigue of the experimental group compared to the control group (*F* = 26.17, *P* < 0.001). Specifically, the experimental group showed decreased stress scores from the baseline to 3 weeks, which continued from 3 to 4 weeks. In contrast, increased stress scores were observed in control group from baseline to 3 weeks, with a much greater increase occurring during the next week (Table 4).

### 3.4. Heart Rate Variability

Over the time frame, HRV of both experimental and control groups was likely to be changed in parallel to 3 weeks after baseline; however, it did not between 3 weeks and 4 weeks. There were significant differences over time of the experimental group compared to the control group in the SDDN (*F* = 6.68, *P* = 0.002), TP (*F* = 5.29, *P* = 0.006), LF (*F* = 4.58, *P* = 0.012), and HF (*F* = 3.71, *P* = 0.026). SDDN, TP, LF, and HF of the experimental group increased from baseline to 3 weeks, and this rise continued from 3 to 4 weeks. The control group showed an increased SDDN, TP, and LF from 3 to 4 weeks; however, it decreased over the next week. The LF of the control group decreased from the baseline to 3 weeks; however, it rose to near baseline value over the next week (see Table 5).

## 4. Discussion

To the best of our knowledge, this is the first study to evaluate the effects of guided imagery on stress and fatigue in patients undergoing radioactive iodine therapy after thyroidectomy. Patients in the experimental group listened to a guided imagery CD once a day for 4 weeks (total = 28 sessions). They were evaluated immediately before they began radioactive iodine therapy when they completed 21 intervention sessions and 1 week after the therapy when they completed 28 sessions. Our findings revealed that guided imagery can be considered an effective intervention to control stress and fatigue among thyroid patients undergoing radioactive iodine therapy after surgery. 

Although there is no clear explanation of how guided imagery brings about positive physical changes, one possible explanation has been provided by Bedford, who believes that perceptual processes are involved in imagery [32]. The combined effect of imagery on physical healing is simply another example of cross-modal adaptation in perception. Guided imagery for healing often includes positive content of images, giving a sense of wellbeing. The positive content of these images produces the conflict with body system, which in contrast indicates something abnormal, such as a cancer cell. Further study should focus on exploring the mechanism behind the effects of guided imagery on physical healing to ensure a better understanding of its importance and provide more effective clinical implementation of guided imagery.

Because guided imagery has not been previously conducted in patients with thyroid cancer undergoing radioactive iodine therapy after surgery, we compared the results of this study with those of other studies examining the effects of guide imagery intervention in various clinical settings. Guided imagery has been shown to decrease stress in women with fibromyalgia [11], hospitalized pregnant women [33], and patients with inflammatory bowel disease [34]. Kolcaba and Fox reported that guided imagery was effective at increasing the comfort of women undergoing radiation therapy for early stage breast cancer, especially in the first three weeks of therapy [15].

Nonpharmacological measures such as mindfulness or massage have also been tested as supporting cancer care. Bränström et al. tested the effects of mindfulness and found that although participants underwent an 8-week mindfulness training course, there were no differences in stress between an experimental group and a control group [35]. Krohn et al. investigated the effects of massage on stress in breast cancer patients and found no significant change after therapy [36]. Kwekkeboom et al. evaluated the feasibility of a patient-controlled cognitive behavioral intervention consisting of relaxation, guided imagery, and nature sound recordings for fatigue during treatment for advanced cancer and found that intervention reduced fatigue [37]. These discrepancies may be because guided imagery is easier to learn and practice than mindfulness, and massage is usually not easy to self-apply.

Guided imagery is a simple, easily taught, and acquired intervention, but it has less been concerned as an intervention methods in cancer patients. Roffe et al. reviewed six studies of guided imagery as an adjuvant cancer therapy and concluded that guided imagery may be psychosupportive and increase comfort [38]. Most recent studies investigating the effects of guided imagery in oncology were conducted among breast cancer patients to examine the effectiveness on quality of life [16] or on biological markers in cancer [39]. Accordingly, more studies are necessary to examine the efficacy of guided imagery in a more diverse setting. 

We evaluated the first posttest on the day participants were admitted to the hospital immediately before they began radioactive iodine therapy because patients undergoing the therapy experienced the highest level of stress due to the therapy and remaining in isolation [30], as well as a high level of fatigue due to the need to stop taking thyroid hormone for the therapy [29]. The second posttest was conducted 1 week after the radiotherapy since patients had the highest level of fatigue as side effects of radioactive iodine therapy [29], which resulted in high levels of stress at this time [31]. Kolcaba and Fox suggested that there may be a decrease in the effective size of guided imagery on certain symptoms at 18 weeks as participants lost interest [15]. However, the long-term efficacy of guided imagery intervention on stress and fatigue was not the subject of this study. Further studies investigating the long-term effects of guided imagery intervention on stress and fatigue among patients undergoing radioactive iodine therapy are recommended.

HRV is a commonly used tool for assessing autonomic function of the heart [28], as well as stress [26] and fatigue [27]. Previous studies demonstrated that HRV is significantly lower in cancer patients than the healthy population [40], and decreased HRV was associated with significantly shorter survival in cancer patients [41]. In the present study, SDNN and TP were improved with 4 weeks of guided imagery intervention, indicating that autonomic function of the heart was improved by guided imagery intervention. 

In this study, the levels HRV of the both experimental and control groups change in parallel before the radioactive iodine therapy; however they did not over the next week. After stress-related treatment, the levels of SDDN, TP, and LF in the experimental group had increased whereas there were decreases in those levels in the control group. We guess the reason why dramatic changes appeared 3 weeks after treatment is because the guided imagery is effective more than 3 weeks of intervention period [19, 20]. 

The results of previous studies also suggest that guided imagery influenced HRV in coronary artery patients. Kong explored the effects of guided imagery intervention among coronary artery patients and found that SDNN, TP, and HF were increased [42]. Chuang et al. applied music therapy to breast cancer patients and reported that SDNN, TP, HF, and LF increased [43]. These results are consistent with the results of our study. However, Asher et al. reported that a guided relaxation program composed of deep breathing exercises and guided imaging did not result in significant differences in HF and LF among cancer patients [44]. One possible reason for the differences between our results and those reported by other studies may be the participant characteristics. For example, Asher did not control the medication, while our study excluded subjects who had any medication. The other possible reason may be the time of postintervention measurement. Most previous studies measured the effect of treatment on HRV without any stress-related events in the middle of study periods. We guess the possibility of the effect of radioactive iodine on autonomic nervous system. More research is required to evaluate this inconsistent result and better understand the effects of guided imagery on HRV in various populations.

It should be noted that this study has some limitations. Specifically, participants in this study were from one hospital and may not have been representative of the general population with thyroid cancer. We sent text messages to participants in the experimental group to remind them of practicing guided imagery every day at the same time and made weekly calls to determine if they were able to practice imagery consistently; however, we are not sure if these activities influenced participant stress and fatigue. Moreover, it is unclear if the fact that the intervention group was doing something repetitive every day led to the differences or if it was the visualization-based relaxation exercises. We could randomly assign in this study. Therefore, we suggest more research is needed with a more comparable control group and random assignments as the next step.

In conclusion, although being diagnosed with cancer results in heavy emotional stress and fatigue, there have been few intervention strategies considered. The results of this study showed that the experimental group experienced lower stress and fatigue than the control group. We recommend that health providers consider guided imagery to reduce stress and fatigue when patients undergo thyroidectomy.

## Figures and Tables

**Table 1:** General characteristics of experimental and control groups (*N* = 84).

Characteristics	Total (*n* = 84)	Exp. (*n* = 44)	Con. (*n* = 40)	*χ*²	*P *
	*n* (%)	*n* (%)	*n* (%)		
Gender					
Male	19 (22.6)	8 (18.2)	11 (27.5)	1.039	0.308
Female	65 (77.4)	36 (81.8)	29 (72.5)		
Age (yr)					
≤30	5 (6.0)	4 (9.1)	1 (2.5)	3.933	0.415
31–40	14 (16.7)	8 (18.2)	6 (15.0)		
41–50	24 (28.6)	14 (31.8)	10 (25.0)		
51–60	28 (33.3)	11 (25.0)	17 (42.5)		
≥61	13 (15.4)	7 (15.9)	6 (15.0)		
Education					
Under middle school	21 (25.0)	10 (22.7)	11 (27.5)	3.497	0.174
High school	25 (29.8)	10 (22.7)	15 (37.5)		
Over college	38 (45.2)	24 (54.6)	14 (35.0)		
Currently employed					
Yes	43 (51.2)	20 (45.5)	23 (57.5)	1.217	0.270
No	41 (48.8)	24 (54.5)	17 (42.5)		
Marital status					
Single	5 (6.0)	3 (6.8)	2 (5.0)	0.525	0.913
Married	69 (82.1)	35 (79.5)	34 (85.0)		
Widowed	8 (9.5)	5 (11.4)	3 (7.5)		
Separated	2 (2.4)	1 (2.3)	1 (2.5)		
Religious					
Yes	40 (47.6)	18 (40.9)	22 (55.0)	1.668	0.197
No	44 (52.4)	26 (59.1)	18 (45.0)		
Perceived economic status					
High	8 (9.5)	5 (11.4)	3 (7.5)	0.368	0.832
Middle	70 (83.3)	36 (81.8)	34 (85.0)		
Low	6 (7.2)	3 (6.8)	3 (7.5)		
Amount of radioactive iodine (mCi)					
100	56 (66.7)	26 (59.1)	30 (75.0)	2.577	0.276
150	21 (25.0)	14 (31.8)	7 (17.5)		
≥180	7 (8.3)	4 (9.1)	3 (7.5)		

**Table 2:** Homogeneity between experimental and control sroups (*N* = 84).

Variables	Exp. (*n* = 44)	Con. (*n* = 40)	*t*	*P*
	M ± SD	M ± SD		
Stress	28.23 ± 15.40	29.05 ± 12.77	0.265	0.792
Fatigue	91.52 ± 42.84	92.30 ± 29.60	0.096	0.924
Heart rate variability				
Standard deviation of all NN intervals (SDNN)	23.06 ± 8.00	25.94 ± 11.77	1.322	0.190
Total power (TP)	5.84 ± 0.76	5.95 ± 0.90	0.585	0.560
Low frequency (LF)	4.41 ± 0.96	4.54 ± 1.16	0.583	0.561
High frequency (HF)	4.15 ± 1.15	4.21 ± 1.22	0.189	0.851

**Table 3:** Stress between experimental and control groups according to time intervals (*N* = 84).

Time	Exp. (*n* = 44)	Con. (*n* = 40)	Source	*F*	*P*
	M ± SD	M ± SD			
Baseline	28.23 ± 15.40	29.05 ± 12.77	Group	28.71	<0.001
3 weeks later	22.16 ± 12.00	38.88 ± 14.43	Time	0.94	0.394
4 weeks later	18.07 ± 12.90	38.83 ± 16.70	Group ∗ Time	19.68	<0.001

**Table 4:** Fatigue between experimental and control groups according to time intervals (*N* = 84).

Time	Exp. (*n* = 44)	Con. (*n* = 40)	Source	*F*	*P*
	M ± SD	M ± SD			
Baseline	91.52 ± 42.84	92.30 ± 29.61	Group	25.99	<0.001
3 weeks later	70.77 ± 34.78	107.07 ± 33.14	Time	0.88	0.416
4 weeks later	61.75 ± 44.14	128.15 ± 46.93	Group ∗ Time	26.17	<0.001

**Table 5:** Heart rate variability between experimental and control groups according to time intervals (*N* = 84).

HRV	Time	Exp. (*n* = 44)	Con. (*n* = 40)	Source	*F*	*P*
		M ± SD	M ± SD			
SDDN	Baseline	23.06 ± 8.00	25.94 ± 11.77	Group	0.73	0.397
	3 weeks later	29.30 ± 10.57	28.69 ± 10.76	Time	9.74	<0.001
	4 weeks later	32.82 ± 9.85	26.50 ± 7.98	Group ∗ Time	6.68	0.002

TP	Baseline	5.84 ± 0.76	5.95 ± 0.90	Group	2.24	0.138
	3 weeks later	6.35 ± 0.80	6.25 ± 0.86	Time	10.82	<0.001
	4 weeks later	6.60 ± 0.67	6.05 ± 0.64	Group ∗ Time	5.29	0.006

LF	Baseline	4.41 ± 0.96	4.54 ± 1.16	Group	1.11	0.296
	3 weeks later	4.91 ± 0.90	4.87 ± 1.02	Time	5.57	0.005
	4 weeks later	5.05 ± 0.99	4.45 ± 0.80	Group ∗ Time	4.58	0.012

HF	Baseline	4.16 ± 1.15	4.21 ± 1.22	Group	4.11	0.046
	3 weeks later	4.40 ± 1.05	4.03 ± 1.07	Time	4.82	0.009
	4 weeks later	4.95 ± 1.01	4.22 ± 1.08	Group ∗ Time	3.71	0.026

SDNN: standard deviation of all NN intervals; TP: total power; LF: low frequency; HF: high frequency.
